# Intravenous perampanel for early post-traumatic seizure prophylaxis across a wide range of traumatic brain injury severity: Potential for low incidence and favorable outcomes in a retrospective cohort

**DOI:** 10.1016/j.bas.2026.106276

**Published:** 2026-08-11

**Authors:** Yuichi Fujiyama, Eiichi Suehiro, Kohei Haji, Koki Okazaki, Hideyuki Ishihara

**Affiliations:** a Department of Neurosurgery, Kenwakai Otemachi Hospital, Kitakyushu, Fukuoka, Japan; b Department of Emergency Medicine, International University of Health and Welfare, School of Medicine, Narita, Chiba, Japan; c Department of Neurosurgery, National Organization Kanmon Medical Center, Shimonoseki, Yamaguchi, Japan; d Department of Neurosurgery, Yamaguchi Graduate School of Medicine, Ube, Yamaguchi, Japan

**Keywords:** Traumatic brain injury, Early post-traumatic seizures, Perampanel, AMPA receptor antagonist, Seizure prophylaxis, Neuroprotection

## Abstract

**Introduction:**

Early post-traumatic seizures (EPTS) are a significant complication of traumatic brain injury (TBI) that impairs neurological recovery. Perampanel (PER), a selective AMPA receptor antagonist, is a promising prophylactic agent with neuroprotective potential, but its intravenous use in mixed-severity TBI remains unexplored.

**Research question:**

We evaluated EPTS incidence and clinical outcomes in TBI patients of all severities receiving intravenous PER as the sole prophylactic agent within 12 h of injury.

**Material and methods:**

This single-center, retrospective study included 75 patients with TBI (GCS 3-15) who received intravenous PER 2 mg within 12 h of injury (May 2024-December 2025). The primary outcome was EPTS incidence within 7 days; secondary outcomes included seizure characteristics, GOS-E, late seizures, mortality, and adverse events.

**Results:**

EPTS occurred in five patients (6.7%; 95% CI, 2.88%-14.68%); all seizures were convulsive, with none identified as non-convulsive. EPTS patients had lower GCS scores (median 4 vs. 12; p = 0.03), longer mechanical ventilation (p = 0.024), extended ICU/HCU stays (p = 0.031), and lower GOS-E at discharge and 3 months (favorable: 40% vs. 84.4%; p = 0.043); 81.2% of 69 followed patients achieved favorable GOS-E at 3 months. Late seizures occurred in 4.3%, and one patient (1.3%) had a PER-related adverse event, with no psychiatric events.

**Discussion and conclusion:**

Intravenous PER within 12 h of TBI was associated with low EPTS incidence, favorable outcomes, and few adverse events. Given the retrospective, single-arm design and modest sample size, these preliminary, hypothesis-generating findings warrant evaluation in the ongoing PEACE-TBI trial.

## Highlights

• EPTS occurred in 6.7% of TBI patients (GCS 3-15) given intravenous perampanel.
• 81.2% of patients achieved a favorable GOS-E outcome at 3 months.
• All seizures were convulsive; none identified as non-convulsive.
• A PER-related adverse event occurred in only 1.3% of patients.
• Findings are hypothesis-generating, warranting evaluation in the PEACE-TBI trial.

## 1 Introduction

Traumatic brain injury (TBI) constitutes a primary cause of morbidity and mortality worldwide, posing a significant public health challenge across all age groups. Early post-traumatic seizures (EPTS), defined as seizures occurring within 7 days of injury, are a clinically significant complication of TBI. EPTS incidence varies depending on the patient population, injury severity, and seizure definition, with reported rates ranging from 0.4% to 10.5% in patients with moderate to severe TBI(1). EPTS is associated with secondary neurological injury, extended intensive care unit (ICU) stays, and poorer functional outcomes, highlighting the critical need for effective prophylactic strategies (Laing et al., 2022; Xu et al., 2017).

Levetiracetam (LEV) has been extensively adopted as a first-line agent for EPTS prophylaxis, largely supplanting phenytoin due to its more favorable tolerability profile (Temkin et al., 1990). Nevertheless, the mechanism of action of LEV, which involves modulation of synaptic vesicle protein 2A (SV2A), does not specifically address the glutamate-mediated excitotoxic cascade that is pivotal in secondary brain injury following TBI. Perampanel (PER), a selective non-competitive antagonist of the α-amino-3-hydroxy-5-methyl-4-isoxazole propionic acid (AMPA) receptor, offers a mechanistically distinct approach by directly inhibiting AMPA receptor-mediated excitatory neurotransmission (Chen et al., 2017; Hou et al., 2025). In preclinical TBI models, PER has been reported to reduce neuronal apoptosis, brain edema, and proinflammatory cytokine expression, suggesting a potential neuroprotective effect beyond seizure suppression (Chen et al., 2017; Aida et al., 2020). However, these mechanistic findings have not yet been directly demonstrated in clinical studies of TBI, including the present study.

Despite these theoretical advantages, clinical evidence supporting PER use in TBI remains limited. Two recent retrospective studies have demonstrated a significantly lower EPTS incidence in patients with PER than in those receiving LEV – 1.8% vs. 15.9% in severe TBI (Onda et al.) and 7.7% vs. 42.1% in moderate-to-severe TBI (Nakao et al.) – alongside more favorable neurological outcomes (Onda et al., 2025; Nakao et al., 2025). However, these studies were restricted to populations with moderate-to-severe TBI only. To our knowledge, no prior study has reported the outcomes of intravenous PER administration in a cohort with mild to severe TBI. Therefore, the present study is intended as a descriptive, hypothesis-generating report that complements the existing literature, rather than as a demonstration of PER efficacy.

The primary aim of this study was to assess the incidence of EPTS and clinical outcomes in a selected single-center cohort of patients with TBI spanning a wide range of injury severities who received intravenous PER as the sole prophylactic agent within 12 h post-injury. The secondary objectives included characterization of seizure profiles, evaluation of late seizure occurrence, mortality rates, and safety profile of intravenous PER in the acute TBI context.

## 2 Materials and methods

### 2.1 Study design and setting

This single-center, retrospective observational study was conducted at the Department of Neurosurgery, Kenwakai-Otemachi Hospital, Fukuoka, Japan. The study protocol was approved by the Institutional Ethics Committee (Approval Number: 23007), and the requirement for individual informed consent was waived because of the study's retrospective nature. An opt-out method was used, in which patients and their families were informed of the study through public notices posted at the institution, and those who chose not to participate were excluded from the study. This study was conducted in accordance with the Declaration of Helsinki and its subsequent amendments.

### 2.2 Patient selection

We conducted a retrospective review of the medical records of all patients with TBI who were admitted to our institution between May 2024 and December 2025. Eligibility for inclusion required that patients received intravenous PER for EPTS prophylaxis within 12 h of injury.

**Fig. 1:**
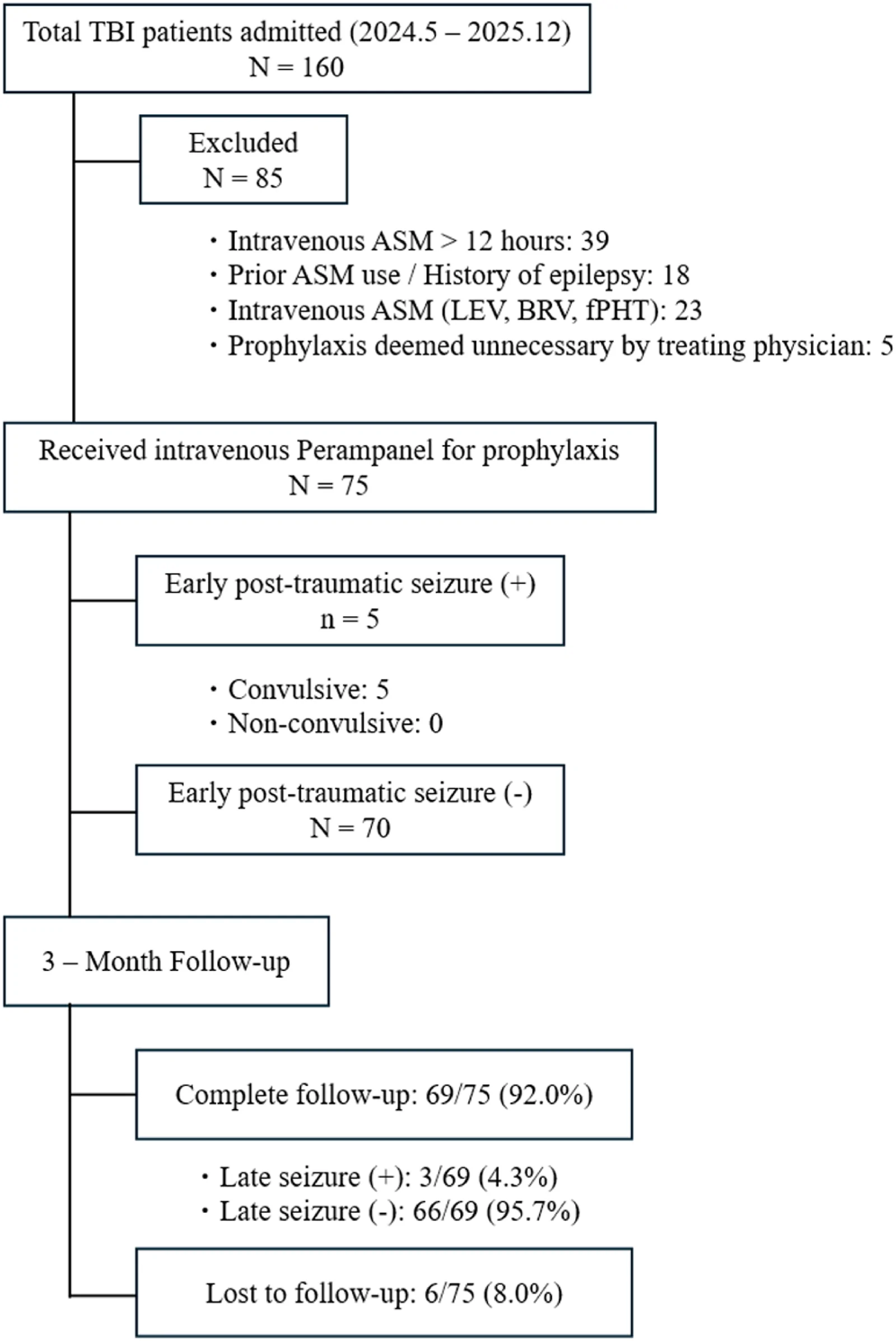
Study Flow Diagram Of 160 traumatic brain injury (TBI) patients admitted during the study period, 85 were excluded. The remaining 75 patients received intravenous Perampanel for early post-traumatic seizure (EPTS) prophylaxis. EPTS occurred in five patients (6.7%), all convulsive. 3-month follow-up was completed for 69 patients (92.0%), and late seizures were observed in three patients (4.3%). ASM: Antiseizure medicine, LEV: Levetiracetam, BRV: Brivaracetam, fPHT: Fosphenytoin.

The exclusion criteria were as follows (Laing et al., 2022): initiation of intravenous ASM > 12 h post-injury (Xu et al., 2017); prior use of ASM or a documented history of epilepsy (Temkin et al., 1990); administration of other intravenous ASMs, including Levetiracetam (LEV), Brivaracetam (BRV), or fosphenytoin (fPHT); and (Chen et al., 2017) prophylaxis deemed unnecessary by the treating physician. Because eligibility depended in part on the treating physician's judgment that seizure prophylaxis was clinically indicated, patient selection may be susceptible to selection bias, which is addressed further in the limitations section. The patient selection process is shown in Fig. 1.

### 2.3 Perampanel administration protocol

All patients were administered intravenous PER at an initial dose of 2 mg within 12 h of injury. Dose adjustments according to body weight, age, or hepatic function were not systematically applied, as PER was administered at a fixed initial dose of 2 mg, regardless of these factors. Intravenous PER was infused over 30 min via a peripheral venous line. For patients requiring enteral access, PER administration was transitioned from intravenous to oral (2 mg) after 1 – 2 days, contingent on gastrointestinal function. The prophylactic regimen was maintained for 7 days.

### 2.4 Management of breakthrough seizures

In patients who developed EPTS during the prophylactic period, PER was continued at 2 mg, with dose escalation to a maintenance level of 4 mg, two weeks after the initial dose. This post-seizure management protocol was distinct from the prophylactic regimen described above.

### 2.5 Definition of early post-traumatic seizures

EPTS was defined as seizures occurring within 7 days of injury, confirmed by standard electroencephalography (EEG) and the American Clinical Neurophysiology Society (ACNS) Standardized Critical Care EEG Terminology (Temkin et al., 1990; Hirsch et al., 2021; Beghi et al., 2010). Non-convulsive status epilepticus (NCSE) was diagnosed according to the Salzburg Consensus Criteria (Leitinger et al., 2016). Standard EEG was performed when clinical indicators of seizure activity were observed, including convulsive seizures, eye deviation, or unexplained deterioration of consciousness (Claassen et al., 2004). For suspected non-convulsive seizures, bedside SedLine brain function monitoring (Masimo Corporation, Irvine, CA, USA) was used as an adjunct, with standard EEG obtained when abnormalities were identified. SedLine monitoring was reviewed for asymmetry in the Density Spectral Array between the hemispheres and for abnormal EEG waveforms, such as spikes or slow waves. Confirmatory standard EEG was performed when these findings were observed. However, no predefined quantitative threshold (e.g., degree of asymmetry or spike frequency) was established a priori; the decision to obtain a confirmatory EEG was based on the qualitative judgment of the treating physician, which may limit the reproducibility of this screening approach. Continuous EEG (cEEG) was not available during the study period.

### 2.6 Data collection

Baseline demographic and clinical data were collected from medical records, including age, sex, mechanism of injury, initial Glasgow Coma Scale (GCS) score, and TBI severity, which was categorized as mild (GCS 13-15), moderate (GCS 9-12), or severe (GCS 3-8). Additionally, head Abbreviated Injury Scale (AIS) score, computed tomography (CT) findings, including the Marshall CT classification (Marshall et al., 1991), surgical interventions, antithrombotic therapy use, and comorbidities were documented. The Marshall CT classification was dichotomized into diffuse injury (Categories II-III) and mass lesions (evacuated or non-evacuated) for analysis.

### 2.7 Outcome measures

The primary outcome measured was the incidence of EPTS within 7 days of injury. Secondary outcomes included seizure characteristics, including seizure type, time to first seizure, and the occurrence of status epilepticus, defined as continuous seizure activity lasting ≥5 min or recurrent seizures without recovery of consciousness between episodes (Lowenstein et al., 1999; Trinka et al., 2015). Clinical outcomes were assessed using the duration of mechanical ventilation (MV), length of stay in the intensive care unit (ICU) or high-care unit (HCU), and the Glasgow Outcome Scale – Extended (GOS-E) score at discharge and at the three-month follow-up. GOS-E scores of 5-8 were defined as favorable outcomes (Wilson et al., 1998). The GOS-E assessment at discharge was performed by the treating neurosurgeon using a structured interview; the assessor was not blinded to the EPTS status. The GOS-E assessment at 3-month was also performed by the treating neurosurgeon on an outpatient basis. However, if the patient was hospitalized at another hospital, the neurosurgeon would call that hospital and conduct a telephone interview with the treating physician to perform the assessment. For six patients who were lost to follow-up, vital status and outcome data could not be obtained.

Additionally, the incidence of late seizures, defined as those occurring > 7 days post-injury during the 3-month follow-up period, mortality rates (both in-hospital and at three months), and adverse events associated with PER administration were evaluated. Late seizures were identified through outpatient follow-up visits, telephone contact when in-person visits were not feasible, and a review of medical records.

Adverse events were graded according to the Common Terminology Criteria for Adverse Events, Version 5.0 (CTCAE v5.0), a standardized grading scale ranging from grade 1 (mild) to grade 5 (death related to adverse event) (National Cancer Institute, 2017). The relatedness of adverse events to PER was determined by the treating physician based on temporal association, exclusion of alternative causes, and the known pharmacological effects of PER.

### 2.8 Statistical analysis

Statistical analyses were conducted using GraphPad Prism (version 10.6.1, GraphPad Software, San Diego, CA, USA). Continuous variables are expressed as medians with interquartile ranges (IQR) and were compared between the EPTS (+) and EPTS (−) groups using the Mann-Whitney *U* test. Categorical variables are presented as counts and percentages and were compared using Fisher's exact test. The 95% confidence interval (CI) for the EPTS incidence rate was calculated using the Wilson score method. Seizure-free survival was estimated using the Kaplan-Meier method. A two-sided p-value of <0.05 was considered to be statistically significant. For the Kaplan-Meier analysis of seizure-free survival, patients were censored at the end of the 7-day observation period if no seizure occurred; no patients died or were discharged/transferred during this 7-day period; therefore, competing events did not affect the censoring structure of this analysis.

## 3 Results

### 3.1 Patient selection and demographics

From May 2024 to December 2025, our institution admitted 160 patients with TBI to the neurosurgery department for treatment. After excluding 85 patients who met the exclusion criteria: intravenous ASM initiated > 12 h post-injury (n = 39), prior ASM use or documented epilepsy (n = 18), administration of other intravenous ASMs including LEV, BRV, or fPHT (n = 23), and prophylaxis deemed unnecessary by the treating physician (n = 5), the final analysis included 75 patients who received intravenous PER for EPTS prophylaxis (Fig. 1). Because patients for whom prophylaxis was considered unnecessary were excluded, this cohort represents a selected group of PER-treated patients with a wide range of TBI severities, rather than an unselected or fully representative TBI population.

### 3.2 Baseline characteristics

Table 1 summarizes the baseline characteristics of the patients. The median age was 75 years (interquartile range [IQR], 55-82), and 38 patients (50.7%) were aged ≥ 75 years. Fifty (66.7%) patients were men. The most common injury mechanism was fall from ground level (n = 42, 56.0%), followed by traffic accidents (n = 19, 25.3%) and fall from height (n = 13, 17.3%). The median initial GCS score was 12 (IQR, 10-14). Fifteen patients (20.0%) had severe TBI (GCS 3-8), 30 (40.0%) had moderate TBI (GCS 9-12), and 30 (40.0%) had mild TBI (GCS 13-15). Cerebral contusion was the most frequent imaging finding, present in 43 patients (57.3%), followed by acute subdural hematoma (n = 25, 33.3%). One patient (1.3%) had a documented history of alcohol dependence.

**Table 1:** Baseline characteristics of all patients.

	Total (N = 75)	EPTS (+) (n = 5)	EPTS (−) (n = 70)	p-value
**Demographics**				
Age (years)	75 (55-82)	79 (52-86)	74 (54-80)	0.6223
Injury mechanism				0.9637
Fall from ground level	42 (56.0%)	2 (40%)	40 (57.1%)	
Fall from height	13 (17.3%)	1 (20%)	12 (17.1%)	
Traffic accident	19 (25.3%)	2 (40%)	17 (24.3%)	
Other	1 (1.3%)	0 (0%)	1 (1.4%)	
**Clinical severity**				
Initial GCS	12 (10-14)	4 (4-12)	12 (10-14)	0.0297 *
GCS category				0.0681
13 - 15 (Mild)	30 (40%)	1 (20%)	29 (41.4%)	
9 - 12 (Moderate)	30 (40%)	1 (20%)	29 (41.4%)	
3 - 8 (Severe)	15 (20%)	3 (60%)	12 (17.1%)	
Head AIS	3 (2-5)	4 (3-4)	3 (2-5)	0.6571
Marshall CT classification, n (%)				0.1647
Diffuse injury (II - III)	52 (69.3%)	2 (40.0%)	50 (71.4%)	
Mass lesion (evacuated or non-evacuated)	23 (30.7%)	3 (60.0%)	20 (28.6%)	
**Image findings**				
Cerebral contusion	43 (57.3%)	5 (100%)	38 (54.3%)	0.0674
Contusion volume (cm^3^)	3.01 (1.38-7.30)	9.58 (6.90-31.11)	2.56 (1.27-4.53)	0.0039 **
Bilateral lesion, n (%)	7 (16.3%)	1 (20.0%)	6 (15.8%)	>0.9999
Location, n (%)				>0.9999
Frontal lobe	24 (55.8%)	3 (60.0%)	21 (55.3%)	
Temporal lobe	12 (27.9%)	1 (20.0%)	11 (28.9%)	
Other	7 (16.3%)	1 (20.0%)	6 (15.8%)	
Acute subdural hematoma	25 (33.3%)	0	25 (35.7%)	0.1625
Acute epidural hematoma	5 (6.7%)	0	5 (7.1%)	>0.9999
Traumatic SAH	1 (1.3%)	0	1 (1.4%)	>0.9999
Diffuse axonal injury	1 (1.3%)	0	1 (1.4%)	>0.9999
**Comorbidities and Clinical Management**				
Alcohol dependence, n (%)	1 (1.3%)	1 (20%)	0	0.0667
Antithrombotic therapy, n (%)	10 (13.3%)	0 (0%)	10 (14.3%)	>0.9999
Surgical intervention, n (%)	20 (26.7%)	2 (40.0%)	18 (25.7%)	0.6047

Baseline characteristics of all patients. Data are presented as median (interquartile range, IQR) or as number (percentages, %). *p < 0.05, **p < 0.01.

No patients experienced hypoxia or hypotension during the acute management period. Sedative agents were administered only in patients requiring surgical intervention (20/20, 100%), and all surgical procedures were performed on the day of injury.

Contusion volume, bilaterality, and lobar location were compared among the 43 patients with cerebral contusion. Lobar location was categorized as frontal lobe, temporal lobe, or other (including parietal lobe, mixed, and cerebellar lesions).

Mann-Whitney *U* test for continuous variables, and Fisher's exact test for categorical variables.

GCS: Glasgow Coma Scale, AIS: Abbreviated Injury Scale, SAH: Subarachnoid hemorrhage, IQR: Interquartile range.

When comparing the EPTS (+) and EPTS (−) groups, the initial GCS score was significantly lower in the EPTS (+) group (median, 4 vs. 12; p = 0.030). The EPTS (+) group also showed a numerically higher proportion of severe TBI (60.0% vs. 17.1%; p = 0.068), cerebral contusion (100% vs. 54.3%; p = 0.067), and alcohol dependence (20.0% vs. 0%; p = 0.067); however, given the small number of EPTS events (n = 5) and multiple unadjusted comparisons, these differences should not be interpreted as being statistically significant. There were no significant differences in age, sex, or injury mechanism between the two groups.

Among the 43 patients with cerebral contusion, the contusion volume was significantly larger in the EPTS (+) group than in the EPTS (−) group (median, 9.58 cm^3^ [IQR, 6.90-31.11] vs. 2.56 cm^3^ [IQR, 1.27-4.53]; p = 0.004, Mann-Whitney *U* test). Bilateral contusion was observed in one of five patients (20.0%) in the EPTS (+) group compared with six of 38 (15.8%) in the EPTS (−) group (p > 0.999, Fisher's exact test). The lobar distribution did not differ significantly between the groups (p > 0.999), with the frontal lobe being the most common site of contusion in both groups (60.0% vs. 55.3%), followed by the temporal lobe (20.0% vs. 28.9%) and other sites (20.0% vs. 15.8%).

### 3.3 Primary outcome: incidence of early post-traumatic seizures

**Fig. 2:**
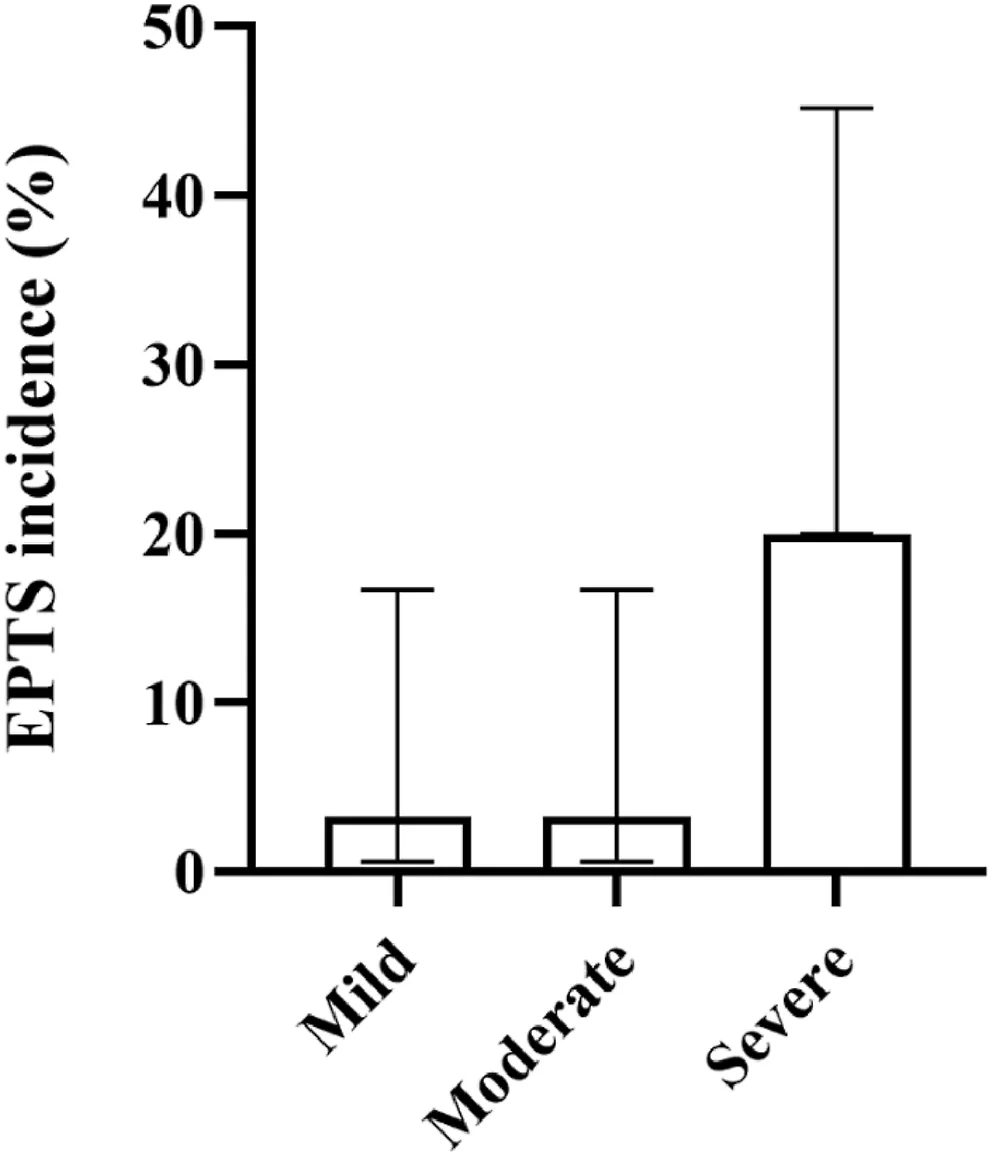
Incidence of early post-traumatic seizures according to TBI severity. The bars present the incidence of EPTS (%) in patients with mild (GCS 13-15), moderate (GCS 9-12), and severe (GCS 3-8) traumatic brain injury. Error bars represent the 95% confidence intervals calculated using the Wilson score method. Although EPTS incidence appeared to increase with injury severity, this trend did not reach statistical significance (chi-square test for trend, χ^2^ = 2.879, df = 1, p = 0.090). TBI, traumatic brain injury; EPTS, early post-traumatic seizures; GCS, Glasgow Coma Scale.

EPTS incidence stratified by TBI severity is presented in Table 2 and Fig. 2. EPTS occurred in 1 of 30 patients with mild TBI (3.3%; 95% CI, 0.6-16.7%), 1 of 30 patients with moderate TBI (3.3%; 95% CI, 0.6-16.7%), and 3 of 15 patients with severe TBI (20.0%; 95% CI, 7.1-45.2%). Although the EPTS incidence numerically increased with injury severity, this trend did not reach statistical significance (chi-square test for trend, χ^2^ = 2.879, df = 1, p = 0.090). This finding suggests that the inclusion of a substantial proportion of patients with mild-to-moderate TBI (40.0% of the cohort) may have contributed to the relatively low overall EPTS of 6.7%, which should be considered when interpreting the primary outcome.

**Table 2:** Incidence of early post-traumatic seizures stratified by TBI severity.

TBI severity	All patients, n	EPTS (+), n	EPTS incidence, %	95% CI (Wilson score)
Mild (GCS 13-15)	30	1	3.3	0.6-16.7
Moderate (GCS 9-12)	30	1	3.3	0.6-16.7
Severe (GCS 3-8)	15	3	20.0	7.1-45.2
**Total**	**75**	**5**	**6.7**	**2.9-14.7**

EPTS incidence is presented as the number and percentage of patients who developed EPTS within each severity category, defined by the initial GCS score at admission (mild, GCS 13-15; moderate, GCS 9-12; and severe, GCS 3-8). The 95% CI for each incidence rate was calculated using the Wilson score method.

EPTS, early post-traumatic seizures; TBI, traumatic brain injury; GCS, Glasgow Coma Scale; CI, confidence interval.

Overall, EPTS occurred in five of the 75 patients (6.7%; 95% confidence interval [CI] [Wilson score], 2.88%-14.68%). All five seizures were convulsive under the seizure detection strategy used in this study. Because continuous EEG monitoring was not available, non-convulsive seizures may have gone undetected rather than being absent. Two patients (40.0%) developed status epilepticus lasting > 5 min. No seizures were identified within the first 24 h of TBI. The median time to the first seizure was 55 h (IQR, 44.5-112.5; range, 39-129 h). Three seizures (60.0%) occurred during the 24-72 h period, and two (40.0%) occurred after 72 h.

### 3.4 Characteristics of EPTS patients

Among the five patients with EPTS, the median age was 79 years (IQR, 52-86), and three (60.0%) were > 75 years (Table 3). Three patients (60.0%) had severe TBI (GCS ≤ 8), and all five patients (100%) had cerebral contusion. Two patients (40.0%) required additional antiseizure medication (levetiracetam), both of whom had status epilepticus (SE). Perampanel was continued in these two patients, and levetiracetam was temporarily added. After titrating perampanel to 4 mg, levetiracetam was discontinued. Three patients (60.0%) required sedation escalation; however, none required intubation or reintubation because of seizure activity.

**Table 3:** Early post-traumatic seizure characteristics.

Incidence		Patient characteristics (n = 5)	
EPTS	5/75 (6.7%)	Age (years)	79 (52 - 86)
95% CI (Wilson score)	2.88% - 14.68%	Age > 75 years	3 (60.0%)
**Timing**		GCS < 8	3 (60.0%)
Time to first EPTS, median (hours)	55 (44.5-112.5)	Cerebral contusion	5 (100%)
Time to first EPTS, range (hours)	39-129	**Management of EPTS**	
Peak incidence period (0 - 24 h)	0 (0%)	Additional ASM required	2 (40.0%)
Peak incidence period (24 - 72 h)	3 (60.0%)	Type of Additional ASM	Levetiracetam 2
Peak incidence period (>72 h)	2 (40.0%)	Sedation escalation	3 (60.0%)
**EPTS type**		Intubation/reintubation	0 (0%)
Convulsive (clinical observation)	5 (100%)		
Non-convulsive (EEG-confirmed only)	0 (0%)		
Status epilepticus (>5 min duration)	2 (40.0%)		

In both patients with status epilepticus, perampanel was continued, and levetiracetam was added temporarily. After uptitration of perampanel to 4 mg, levetiracetam was discontinued.

Data are presented as median (interquartile range, IQR) or as number (percentages, %).

EPTS: Early post-traumatic seizure, CI: Confidence interval, EEG: Electroencephalogram, GCS: Glasgow Coma Scale, SAH: Subarachnoid hemorrhage, ASM: Antiseizure medicine.

### 3.5 Seizure-free survival

**Fig. 3:**
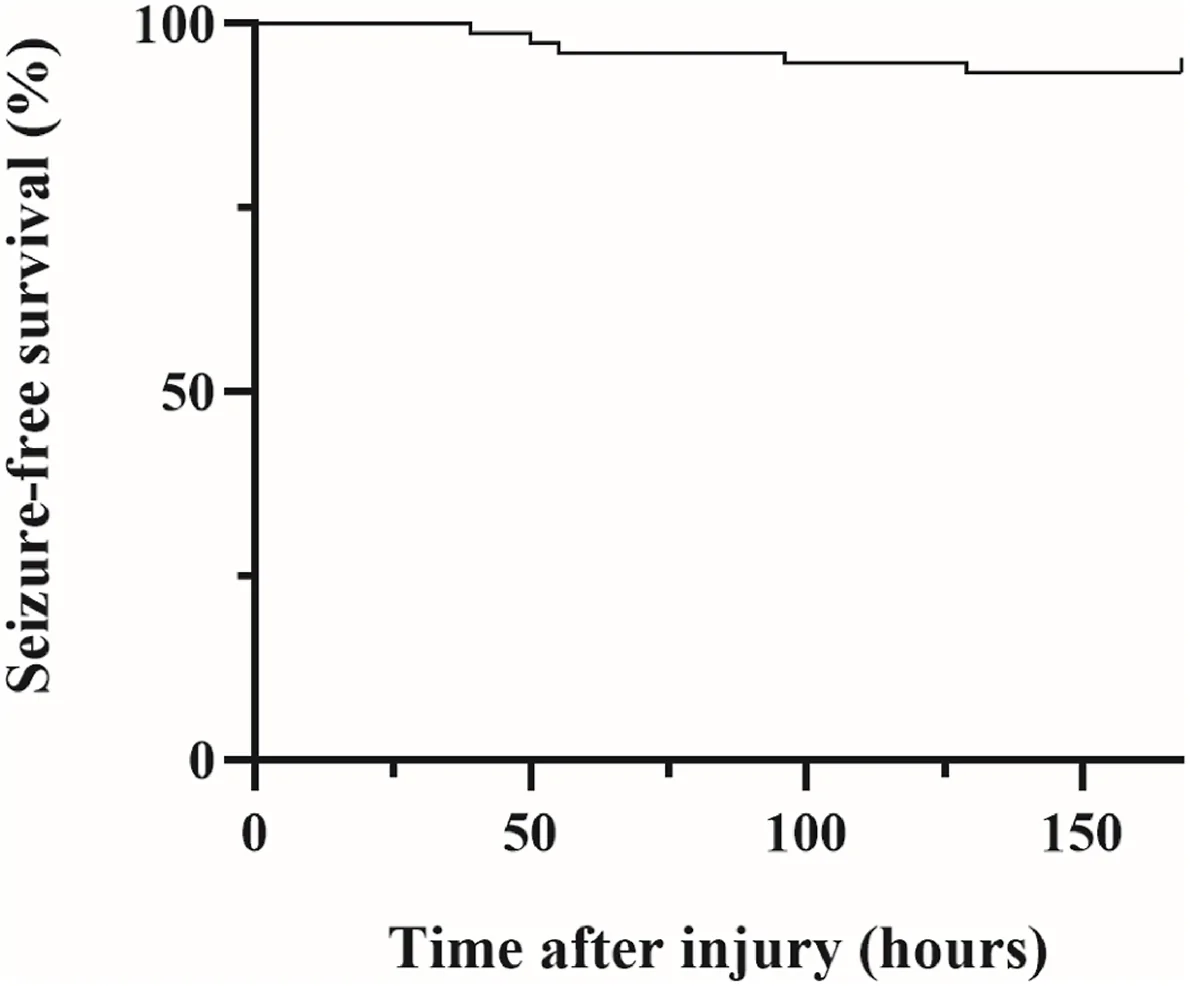
Kaplan-Meier estimate of seizure-free survival. The curve illustrates the likelihood of staying free from early post-traumatic seizures (EPTS) over 7 days (168 h) after injury in 75 patients who received intravenous perampanel. Tick marks denote censored observations. No seizures occurred within the first 39 h. The 7-day seizure-free survival rate was 93.3% (95% CI, 85.3-97.1%). umber at risk: 75 at 0h, 75 at 24h, 74 at 48h, 72 at 72h, 72 at 96h, 70 at 120h, 70 at 168h. CI: Confidence interval.

Kaplan-Meier analysis revealed a 7-day seizure-free survival rate of 93.3% (Fig. 3). The survival curve remained at 100% through the first 39 h, with the earliest seizure onset occurring 39 h after TBI. Subsequently, a stepwise decline was observed, with most events clustering between 39 and 129 h.

### 3.6 Clinical outcomes

The clinical outcomes are shown in Table 4. Overall, 28 of 75 patients (37.3%) required mechanical ventilation (MV), with no significant difference between the EPTS (+) and EPTS (−) groups (60.0% vs. 35.7%; p = 0.356). The subsequent comparison of MV duration was restricted to ventilated patients. Patients with EPTS had significantly longer durations of MV (median 260 h [IQR, 202-288] vs 108 h [IQR,20-190]; p = 0.024) and ICU/HCU stays (median 280 h [IQR, 64-368] vs 55.5 h [IQR, 29.3-117]; p = 0.031) than those without EPTS. Because the EPTS (+) group had lower initial GCS scores and more severe injuries, these differences in MV duration and ICU/HCU stays likely reflect the underlying injury severity rather than a direct consequence of seizure activity. The GOS-E score at discharge was significantly lower in the EPTS (+) group (median 3 [IQR, 3-4] vs 5 [IQR, 4-7]; p = 0.038). The proportion of patients with a favorable GOS-E (Hou et al., 2025; Aida et al., 2020; Onda et al., 2025; Nakao et al., 2025) at discharge did not differ significantly between the groups (20.0% vs. 57.1%; p = 0.170).

**Table 4:** Clinical outcomes.

	Total (N = 75)	EPTS (+) (n = 5)	EPTS (−) (n = 70)	p-value
**Acute Outcome**				
MV duration (hours)	154.5 (28.7-217.5)	260 (202-288)	108 (20-190)	0.0238 *
Patients requiring MV	28 (37.3%)	3 (60.0%)	25 (35.7%)	0.3555
ICU/HCU stay (hours)	57 (31.5-132)	280 (64-368)	55.5 (29.3-117)	0.0305 *
**Outcome at Discharge**				
GOS-E	5 (4-7)	3 (3-4)	5 (4-7)	0.0384 *
95% CI (Wilson score)		0.1% - 12.6%	87.4% - 99.9%	
Favorable GOS-E (5 to 8)	41 (54.7%)	1 (20.0%)	40 (57.1%)	0.1697
**Outcome at 3-Month (n = 69)**				
GOS-E	7 (5-8)	3 (3-7)	7 (5-8)	0.1068
Favorable GOS-E (5 to 8)	56 (81.2%)	2 (40.0%)	54 (84.4%)	0.0429 *
95% CI (Wilson score)		0.6% - 11.7%	87.7% - 99.4%	
Late seizure	3 (4.3%)	0 (0%)	3 (4.7%)	>0.9999
**Mortality**				
In-hospital mortality	7 (9.3%)	0 (0%)	7 (10.0%)	>0.9999
3-Month mortality	8 (11.6%)	0 (0%)	8 (12.5%)	>0.9999

Data are presented as median (interquartile range, IQR) or as number (percentages, %). *p < 0.05.

Mann-Whitney *U* test for continuous variables and Fisher's exact test for categorical variables. All 5 patients in the EPTS (+) group were followed up, but 6 cases in the EPTS (−) group could not be followed up, resulting in a total of 64 cases being followed up.

EPTS: Early post-traumatic seizure, MV: Mechanical ventilation, ICU: Intensive Care Units, HCU: High Care Units, GOS-E: Glasgow Outcome Scale – Extended.

At the 3-month follow-up, 69 of the 75 patients (92.0%) completed the assessment. All five patients in the EPTS (+) group were followed up, whereas six patients in the EPTS (−) group were lost to follow-up. Thus, 64 patients were followed up in the EPTS (−) group. The median GOS-E score at 3 months was 7 [IQR, 5-8] for the overall cohort. A favorable GOS-E was achieved in 56 of the 69 patients (81.2%). However, the EPTS (+) group had a significantly lower favorable GOS-E score (40.0% vs. 84.4%; p = 0.043).

### 3.7 Late seizures

Late seizures (occurring > 7 days post-injury) developed in three of 69 patients (4.3%) during the 3-month follow-up period, ascertained through outpatient follow-up visits and medical record review. No late seizures occurred in the EPTS (+) group (0/5), whereas late seizures were documented in the EPTS (−) group (3/64, 4.7%; p > 0.999).

### 3.8 Mortality

Overall, in-hospital mortality was 9.3% (7/75), with no deaths in the EPTS (+) group (0/5) and seven deaths (10.0%) in the EPTS (−) group (p > 0.999). At the three-month follow-up, the overall mortality was 11.6% (8/69), with no deaths in the EPTS (+) group (0/5) and eight deaths (12.5%) in the EPTS (−) group (p > 0.999).

### 3.9 Safety profile

One of the 75 patients (1.3%) experienced PER-related adverse events (Table 5). This patient experienced transaminase elevation (Common Terminology Criteria for Adverse Events Grade 2), leading to the discontinuation of PER and a switch to levetiracetam. No psychiatric or behavioral adverse events, including irritability, aggression, dizziness, or somnolence, were documented in the reviewed medical records. Similarly, no skin rashes or hematological abnormalities were observed. The discontinuation rate due to adverse events was 1.3% (1/75 patients).

**Table 5:** Adverse events.

	n (%)
Any PER-related adverse event	1 (1.3%)
**Hepatobiliary disorders**	
Transaminase elevation (CTCAE Grade 2)	1 (1.3%)
**Psychiatric and behavioral disorders**	
Irritability/aggression	0 (0%)
Dizziness	0 (0%)
Somnolence	0 (0%)
**Other**	
Skin rash	0 (0%)
Hematological abnormality	0 (0%)
Drug discontinuation due to adverse event	1 (1.3%)

Data are presented as numbers (percentages, %). Perampanel was discontinued and switched to levetiracetam due to transaminase elevation.

PER: Perampanel, CTCAE: Common Terminology Criteria for Adverse Events.

## 4 Discussion

Intravenous PER administered within 12 h post-injury was associated with an EPTS incidence of 6.7% in this cohort. Previously reported PER-based prophylaxis rates in TBI populations range from 15.9% (Onda et al., severe TBI) (Onda et al., 2025) to 42.1% (Nakao et al., moderate-to-severe TBI) (Nakao et al., 2025); however, because this study lacked a comparator group, the present findings cannot be interpreted as demonstrating the superiority of PER over other intravenous ASM, and the observed EPTS incidence lies broadly within the range reported for standard prophylaxis regimens.

Direct comparisons across studies are further constrained by differences in patient populations. In particular, the current cohort comprised individuals with mild-to-severe TBI (GCS 3-15), whereas the previously mentioned studies concentrated solely on moderate-to-severe injuries. The inclusion of patients with mild TBI, who inherently have a lower baseline risk of seizures, likely contributed to the overall reduced EPTS rate observed in this study, as suggested by the severity-stratified analysis (Table 2 and Fig. 2).

Current clinical guidelines recommend either phenytoin (PHT) or levetiracetam (LEV) as first-line agents for EPTS prophylaxis in moderate-to-severe TBI, with LEV increasingly preferred because of its more favorable pharmacokinetic and tolerability profiles compared with PHT (Temkin et al., 1990; Carney et al., 2017). PER was used in the present cohort as an alternative off-label prophylactic agent rather than as a guideline-recommended first-line option. Because this study did not include a concurrent PHT or LEV comparator arm, the present findings cannot establish whether PER offers any advantages over these guideline-based regimens in terms of efficacy and tolerability. Rather, intravenous PER should be positioned as a promising – yet exploratory – component of future risk-stratified TBI seizure prophylaxis algorithms, to be evaluated against PHT and LEV in prospective comparative studies such as PEACE-TBI (Yamamoto et al., 2025).

Notably, among patients with cerebral contusion, the contusion volume was significantly larger in the EPTS (+) group than in the EPTS (−) group, whereas neither bilaterality nor lobar location differed significantly between the groups. This finding suggests that the extent of cortical structural damage, rather than its location or laterality, may be a more relevant determinant of epileptogenic potential in this cohort. Consistent with this, all five patients who developed EPTS had cerebral contusions, and the initial GCS score was significantly lower in the EPTS (+) group than in the EPTS (−) group, consistent with previously identified risk factors for EPTS. Although only three of the five EPTS cases met the criteria for severe TBI (GCS 3-8), the remaining two cases with mild or moderate TBI also had cerebral contusions, suggesting that cortical injury, rather than the GCS category alone, may be a key determinant of seizure risk in this cohort.

The absence of detected non-convulsive seizures (NCSE) in this cohort should be interpreted with caution, as continuous EEG (cEEG) monitoring, the diagnostic standard for identifying subclinical or NCSE activity in critically ill patients (Hirsch et al., 2021), was not available during the study period. Seizure ascertainment relied on clinical observation, adjunctive SedLine monitoring, and confirmatory standard EEG performed only when clinical suspicion arose. Because this detection strategy is inherently less sensitive than continuous monitoring, the true incidence of EPTS in this cohort, particularly in non-convulsive events, may have been underestimated. This limitation is especially relevant given that NCSE has been reported to account for a substantial proportion of EPTS in mechanically ventilated or sedated TBI patients (Claassen et al., 2004).

The relatively low incidence of EPTS observed in this cohort may be partly explained by the distinct mechanism of action of PER, although this study did not directly assess neuroprotection and, therefore, could not establish a causal link. Unlike LEV, which modulates SV2A, PER selectively antagonizes AMPA receptors, the primary mediators of rapid excitatory neurotransmission, thereby directly attenuating the glutamate excitotoxic cascade triggered by TBI(4, 5). This rationale is supported solely by preclinical evidence: in animal models of TBI, PER has been shown to reduce brain edema, neuronal apoptosis, and proinflammatory cytokines (TNF-α, IL-1β) (Chen et al., 2017), and to attenuate cognitive impairment at clinically relevant doses (Aida et al., 2020). These neuroprotective effects have not been demonstrated in the present clinical cohort, which lacked neuroimaging, biological markers, and a comparator group; therefore, the preclinical findings should be regarded as background rationale rather than clinical evidence from this study.

Consistent with prior studies (Onda et al., 2025; Nakao et al., 2025), patients with EPTS experienced prolonged MV, extended ICU/HCU stay, and lower GOS-E scores at both discharge and 3 months post-injury. These findings corroborate the established understanding that EPTS reflects injury severity as much as it causes secondary harm. The lower GCS scores and higher cerebral contusion rate in the EPTS (+) group suggest that poorer outcomes may, at least partly, reflect underlying injury severity rather than seizure activity per se (Laing et al., 2022).

Nevertheless, the overall cohort exhibited favorable neurological outcomes, with 81.2% of patients achieving a favorable GOS-E score at 3 months. This rate is comparable to that reported by Nakao et al. (favorable outcome rate in the PER group: 79.1%) (Nakao et al., 2025) and is notably higher than the rate observed with LEV prophylaxis in the same study (51.3%) (Nakao et al., 2025). However, given the cross-study nature of this comparison and the absence of a concurrent comparator arm in the present study, this observation should be regarded as descriptive rather than evidence of treatment effect, and differences in patient case-mix between studies cannot be excluded as contributing factors.

Late seizures occurred in three patients. No late seizures were observed in the EPTS (+) group; however, this should be interpreted cautiously, given the small size (n = 5). EPTS is the strongest predictor of post-traumatic epilepsy (RR 5.14; 95% CI 2.81-9.41) (Xu et al., 2017), underscoring the need for prolonged neurological monitoring of these patients.

The safety profile of intravenous PER in the current cohort was favorable, with only one patient (1.3%) experiencing a PER-related adverse event, a grade 2 transaminase elevation, that resolved following discontinuation and transition to LEV. Notably, no psychiatric or behavioral adverse events, including irritability, aggression, dizziness, or somnolence, were observed. This finding is clinically significant, given that psychiatric and behavioral adverse events are a well-recognized concern with PER, particularly at higher doses. In a pooled analysis of three phase III randomized controlled trials in patients with partial seizures, Ettinger et al. reported dose-dependent increases in hostility/aggression-related treatment-emergent adverse events, occurring in 2.8% and 6.3% of patients receiving PER at 8 mg and 12 mg per day, respectively, compared to 0.7% in the placebo group (Ettinger et al., 2015). The absence of such events in the present study may reflect both the low prophylactic dose (2 mg) and the confounding effects of reduced consciousness and sedation, which may have masked mild psychiatric symptoms, a limitation further discussed in the Limitations section.

To our knowledge, this is one of the first studies to report real-world experience with intravenous PER as a sole prophylactic agent in a selected cohort with mild to severe TBI severity (GCS 3-15), including mild TBI, a population not addressed by prior studies (Onda et al., 2025; Nakao et al., 2025). The predominance of small, retrospective, single-center studies underscores the need for prospective data. The ongoing PEACE-TBI trial (jRCTs031250067), a phase-II multicenter randomized controlled trial, is designed to assess whether PER improves neurological outcomes in mild-to-moderate TBI(20). The present findings provide real-world support for this evaluation.

## 5 Limitations

This study has several limitations that should be considered when interpreting the findings.

Because it was conducted at a single center with a retrospective design, the generalizability of our findings is limited. It is also worth noting that patients for whom prophylaxis was deemed unnecessary by the treating physician were excluded from the analysis; consequently, this cohort represents a selected group of perampanel-treated patients rather than an unselected TBI population.

The sample size was modest (n = 75), and only five patients developed EPTS. This limited number of events reduced our statistical power to detect meaningful differences in the subgroup analyses or to identify independent predictors of seizure occurrence. Given the few events observed, the multiple unadjusted comparisons performed between the EPTS (+) and EPTS (−) groups are best regarded as exploratory rather than confirmatory.

Another constraint stems from the lack of a comparator group. As a single-arm study, we cannot draw firm conclusions about the efficacy of PER, and our findings should be viewed as hypothesis-generating rather than definitive evidence.

Seizure detection is another area of concern. Continuous EEG (cEEG) monitoring was not available at our institution during the study period, so seizure ascertainment instead relied on clinical observation, supplemented by bedside SedLine brain function monitoring and confirmatory standard EEG when clinically indicated. Non-convulsive seizures account for a substantial proportion of EPTS in critically ill TBI patients, and it is plausible that some such events went undetected under this less sensitive monitoring strategy.

Radiological assessment of cortical injury, including contusion volume, was performed through visual estimation from CT imaging rather than standardized volumetric software; this approach may have introduced measurement variability that a more granular, software-based analysis could avoid in future studies.

Follow-up was also incomplete: six patients were lost to the 3-month follow-up, all of whom belonged to the EPTS (−) group. Combined with the relatively short 3-month window used to ascertain late seizures, this loss to follow-up limits the precision of our reported favorable outcome rate and precludes a fuller characterization of long-term late seizure risk. However, no deaths or discharges occurred during the 7-day observation period used for the Kaplan-Meier analysis of seizure-free survival; therefore, competing risks did not affect that particular analysis.

We collected and analyzed head AIS scores; however, the Injury Severity Score (ISS) and Rotterdam CT score were not evaluated in this study. The overall trauma burden and diffuse injury severity were captured using the Marshall CT classification, which may not offer the same level of granularity as these alternative scoring systems.

Finally, our assessment of behavioral adverse events was complicated by the confounding effects of reduced consciousness and sedation in the acute TBI setting, which may have obscured mild psychiatric symptoms in patients. Addressing this and other limitations noted above will require future prospective studies that incorporate standardized behavioral assessments, volumetric radiological analysis, and extended follow-up, allowing for a more comprehensive evaluation of the neuropsychiatric safety profile and long-term seizure risk associated with PER.

## 6 Conclusion

Intravenous PER administered within 12 h post-injury was associated with a low EPTS incidence of 6.7%, favorable neurological outcomes in 81.2% of patients at 3 months, and an acceptable safety profile, evidenced by a PER-related adverse event rate of 1.3%. These observations should be interpreted as preliminary and hypothesis-generating rather than evidence of efficacy or tolerability, given the retrospective, single-arm design, modest sample size, and inclusion of a substantial proportion of patients with mild TBI, all of which may have contributed to favorable findings independent of any treatment effect. This study offers descriptive real-world evidence for the use of intravenous PER in a selected cohort with a wide range of TBI severities, a population that has been underrepresented in previous research. Prospective multicenter randomized controlled trials, such as the ongoing PEACE-TBI trial, are necessary to determine whether PER is truly effective and well tolerated for EPTS prophylaxis in TBI patients.

## Consent to participate

The requirement for informed consent was waived by the Institutional Ethics Committee due to the retrospective design of the study. An opt-out method was used.

## Author contributions

Y.F.: Conceptualization, data curation, formal analysis, investigation, methodology, writing – original draft, writing – review and editing.

E.S.: Supervision, methodology, writing – review and editing.

K.H.: Investigation, writing – review and editing.

K.O.: Investigation, writing – review and editing.

H.I.: Supervision, methodology, writing – review and editing.

## Ethical considerations

This study was approved by the Institutional Ethics Committee of Kenwakai Otemachi Hospital (Approval Number: 23007). The requirement for individual informed consent was waived because of the retrospective design of the study. An opt-out method was used. This study was conducted in accordance with the Declaration of Helsinki and its subsequent amendments.

## Consent for publication

Not applicable.

## Data availability

The data that support the findings of this study are available from the corresponding author upon reasonable request and subject to appropriate ethical considerations.

During the preparation of this work, the authors used PaperPal and Perplexity AI for language editing and grammar verification. The authors reviewed and edited the output as needed and take full responsibility for the content of the published article.

## Funding statement

The authors received no financial support for the research, authorship, or publication of this article.

## Declaration of competing interests

The authors declare that they have no known competing financial interests or personal relationships that could have appeared to influence the work reported in this paper.
